# Can straw based enrichment treat tail biting in pigs?

**DOI:** 10.18849/ve.v7i3.458

**Published:** 2022-07-20

**Authors:** Abigail Liston+

**Keywords:** ENRICHMENT, INCIDENCE, PIGS, TAIL BITING, TREATMENT

## Abstract

PICO question

Can straw based enrichment be used successfully as a treatment intervention to reduce tail biting injuries in weaner to finisher pigs housed in indoor farming systems?

Clinical bottom line

Category of research question

Treatment

The number and type of study designs reviewed

Three papers were critically reviewed. All three papers answered the PICO question and matched the inclusion criteria for this Knowledge Summary, providing moderate evidence. One non-randomised controlled trial and two randomised controlled trials

Strength of evidence

Moderate

Outcomes reported

Veit et al*. *(2016) found that straw based enrichment can reduce tail biting, similarly, Larson et al. (2018) found straw based enrichment could reduce tail biting, however, other factors contribute more so to reducing tail biting. Haigh et al. (2019) did not find evidence to suggest straw-based enrichment could reduce tail biting. Triggers for tail biting injuries are multifaceted, therefore enrichment alone will not eliminate pen mate manipulation

Conclusion

In view of the strength of evidence and the outcomes from the studies the following conclusions have been made; it is expected that these findings provide enough evidence to encourage farmers to introduce novel straw based enrichment as a treatment measure, however it would be most effective if other husbandry factors could be considered in addition

How to apply this evidence in practice

The application of evidence into practice should take into account multiple factors, not limited to: individual clinical expertise, patient’s circumstances and owners’ values, country, location or clinic where you work, the individual case in front of you, the availability of therapies and resources.

Knowledge Summaries are a resource to help reinforce or inform decision making. They do not override the responsibility or judgement of the practitioner to do what is best for the animal in their care.

## The evidence

Three papers answered the PICO question and matched the inclusion criteria for this Knowledge Summary, providing moderate evidence. One was a non-randomised control trial (Haigh, et al., 2019), two were randomised control trials (Veit et al., 2016; and Larsen et al., 2018). Both randomised control trial studies and the non-randomised control study had moderate or large population sizes and a strong experimental design. All papers focused on subjective measures which may have created variability in the results, however well-established and robust scoring systems were used to reduce any bias in all selected studies. For non-randomised trials, the pigs were not randomly allocated to groups; weight and sex determined their allocation which may have predisposed the pigs to respond differently to enrichment. The observers were not blinded in any of the studies, which means due to confirmation bias they may not have looked for tail injuries as attentively in enriched groups if they believed enrichment would prevent tail biting.

## Summary of the evidence

**Haigh et al. (2019):** 

Population:	Pigs bred from terminal line Pig Improvement Company, born from Large White X Landrace sows, which were born in two replicates of 440 individuals, 7 weeks apart. 75% of the tail was docked at 3 days old, males were uncastrated.
Sample size:	880 pigs.
Intervention details:	Piglets were split into 16 groups of 55 piglets based on weight and sex. At 28 days old half of the piglets (440 split between 8 groups) were provided straw blocks and half were given commercial pig toys. The experiment followed a 2x2 factorial design with sex and enrichment type as the main factors. Two devices (enrichments) were allocated per pen (i.e., 27 pigs per device) at the first and second weaner stage, one device per pen (i.e., 25 pigs per device) in the finisher stage. Straw blocks were cylindrical in shape and provided in a dispenser attached to the wall of the pen. Approximately 10 cm of straw was exposed for use by the pigs in the weaner stage and 7.5 cm of straw exposed in the finisher stage. Two short chains (23 cm) were hung either side of the exposed straw block. There was a continuous supply of straw via the dispensers. During the movement between the first and second weaner pens, individuals were scored for body lesions on the head, shoulders and flank. The tails and ears of all pigs were examined approximately once every 2 weeks starting from 2 weeks after weaning (subject to fitting around farm practices).
Study design:	Non-randomised control trial.
Outcome studied:	Tails and ears of all pigs were examined individually, and behavioural observations of each pig group were conducted fortnightly from weaning through to slaughter. Tail lesion scoring was done using the scoring chart adapted from Hunter et al. (1999).
Main Findings (relevant to PICO question)	More instances of aggression were observed with the toy than with the straw block in the weaner stage (P < 0.05). The results suggested a link between sex and enrichment type in the weaner stage (P < 0.01) as female pigs showed more tail biting behaviour than males when provided with straw blocks (P < 0.05). This is shown by an increase of 0.011 incidence in tail biting in female groups when pigs were given straw blocks as opposed to toys (CI 0.041–0.035 for straw block, 0.030–0.023 for toys). For males, there was a decline in tail biting (incidence reduced by 0.008) when given straw blocks over toys (confidence interval 0.027–0.020 for straw block, 0.037–0.030 for toys). Also, the amount of tail biting was greater in the first and fourth observation week than that on week 7 and 8 (P < 0.05 for all pairwise comparisons). The study found there to be no significant difference in the effectiveness of two commercially available enrichment materials considered appropriate for fully slatted systems, in reducing tail biting.
Limitations:	Inspections of injuries did not always happen every 2 weeks due to logistical issues. Only a total of eight observations were used in analysis, three for the first weaner stage (18 ± 0, 31 ± 4 and 43 ± 4 days post-weaning) and finisher stage (94 ± 4, 119 ± 4 and 137 ± 4 days post-weaning), only two observations during the second weaner stage (55 ± 6 and 73 ± 9 days post-weaning), which may create bias in results. There was no control group. Experimenters were not blinded to treatment group assignment. Two different experimenters were used, which could create subjectivity in scoring.

**Larsen et al. (2018):** 

Population:	Finisher pigs (from 30 kg to slaughter) all from the same herd. Dams of Danavl Yorkshire X Danaval Landrance, inseminated with Danaval Duroc semen.
Sample size:	1,624 pigs.
Intervention details:	Pens were divided into four batches (1, 3 and 4: n = 32 each, batch 2: n = 16). The study ran from June 2015 to November 2016. One weaner section and two finisher sections were used in the study, each containing 16 identical pens. The finisher pens were randomly assigned to one level of each of the three treatments: docked tails (half the length of the original tail), undocked tails, provision of straw, stocking density. Tail scoring commenced when pigs entered the finisher section. Finisher pens with straw were provided with 150 g of straw per pig per day on solid floor. Tail damage was assessed every Monday, Wednesday, and Friday by eight trained observers. It was further supported by daily pen level observations performed by herd level staff. An incidence of tail damage was recorded when at least one pig in a pen had a bleeding tail wound.
Study design:	Randomised control trial.
Outcome studied:	A higher incidence of tail damage was seen in pens without straw compared to pens with straw.
Main Findings (relevant to PICO question)	Finisher pens developed incidences of tail damage mainly in week 1 and the first half of the finisher period. Providing 150 g of straw per pig was an effective preventative measure against tail damage with a more than 2 fold higher hazard of developing tail damage in pens with no straw provided (hazard rate ratios = 2.22, 95% Confidence Interval (1.28–3.86); P <0.01). Tail docking had a larger preventative effect than straw in reducing tail biting injuries. Therefore, this study suggested that straw cannot replace tail docking as a preventative measure. However, lowering stocking density and providing straw can be used to replace tail docking as a preventative measure for tail biting injuries.
Limitations:	Blinding of observers did not occur (it was not possible due to the obvious treatments being used). Statistical analysis to identify the effect of straw was combined with other impacting factors, such as docked / undocked tails and stocking density. The scoring criteria for tail damage was very sensitive, the authors believe the high prevalence (half the pens had recorded tail damage) may be due to mild tail damage definitions and very detailed scoring multiple times per week. This is unlikely to be replicable on working farms and therefore may not be representative of the true impact expected to be seen on farms. The first incidence of tail damage at pen level was recorded, no further incidences were considered. This study does not reflect the likely outcome that multiple incidences of tail damage are likely to occur in a pig’s lifetime, or whether enrichment prevents further incidences. Confidence intervals were not available for this study.

**Veit et al. (2016):** 

Population:	Undocked crossbred piglets (Pietrain X [large white X Landrace]) from 60 litters, housed in 10 batches.
Sample size:	721 piglets.
Intervention details:	Piglets, 2 weeks of age, were divided randomly into three categories: Control group (231 piglets housed without raw material). Dried corn silage group (245 piglets). Alfalfa hay group (245 piglets). In total there were six batches with three pens of 24 piglets and four batches with six pens of 12 piglets. Enrichment was provided twice per day from the second week of life through to the finishing period.
Study design:	Randomised control trial.
Outcome studied:	Each tail was scored regarding tail lesions / tail losses once per week with a four point score (modified by Abriel & Jais, 2013). Tail losses were scored between 0–3. Scoring was carried out by one person. A tail biting outbreak was defined as a point in time when at least one piglet showed moderate tail damage.
Main Findings (relevant to PICO question)	The effect of treatment group had highly significant impact on Class 0 and Class 3 of tail lesions (P < 0.001). Tail lesions in Class 2 tended to be influenced by the treatment group (P = 0.05). There was no significant effect of the treatment group on tail lesions in Class 1 (P = 0.4). At the end of rearing, piglets of all batches had lost their tails to the greatest extent in the control group (48.7%), followed by the alfalfa hay group (45.2%) and the dried corn silage group (41.3%).
Limitations:	The piglet bowl did not ensure every piglet could gain access to the raw material, all at the same time. This competition may have contributed to behavioural disorders. Tail lesions were scored as 0 (original length of tail), 1 (loss of tail tip), 2 (partial loss), or 3 (total loss); short descriptions for each score may have led to ambiguity. Confidence intervals were not available for this study.

## Appraisal, application and reflection

In the Haigh et al. (2019) paper it is necessary to note that all the pigs had 75% of their tails docked prior to commencement of the study which is likely to have influenced the occurrence and severity of tail biting injuries. Tail scoring was done fortnightly in the Haigh et al. (2019) study, allowing time for injuries to potentially heal between scorings. Strengths of the study are the moderate population size plus the study design. To further the credibility of the study a control group could be used, alongside regular scoring. Considering the sample group had docked tails, which could potentially reduce severity of tail biting injuries as opposed to increase, with the addition of the scoring being fortnightly, it would be reasonable to suggest the result, that straw based enrichment did not successfully reduce tail biting incidents, is accurate under these circumstances.

Veit et al. (2016), has a strong study design along with a moderate population size and regular scoring which was completed by the same person, reducing subjectivity. The study found that straw based enrichments reduced the incidences of tail biting in comparison to the control group. However, the scoring chart itself was vague which could have impacted the accuracy of the results.

Larsen et al. (2018) provided a strong level of evidence due to the study design, the number of observations contributing to the results and the presence of a control group to assess the comparative impact of adding straw. However, the study did not assess the ability of straw to stop or reduce tail biting outbreaks once they had started, it focused solely on straw as a preventative measure. Overall, the study found that straw was an effective preventative measure against tail damage, however the greatest preventative measure is still tail docking. There is a suggestion that environmental management, i.e., lower stocking density and straw, could be used in combination to prevent tail biting outbreaks.

It is important to note that preventing tail damage and treating tail damage will involve differing approaches. These studies highlighted whether tail damage was reduced by using straw based enrichments. Overall, it was found that by introducing straw based enrichment, tail damage could be reduced in some cases (Veit et al., 2016). The rearing stage has been highlighted as a time when the most tail damage has been noted and this information could be used in further studies to identify what factors in a pig’s life influence this. In practice, it is expected that farmers should introduce enrichment to reduce tail biting outbreaks alongside targeting other husbandry practices to reduce tail biting further. It may be impractical to expect commercial farms to identify and introduce novel enrichment with sufficient time to prevent an outbreak.

## Methodology Section

**Search Strategy:** 

Databases searched and dates covered:	CAB Abstracts Platform: CAB Direct 1973–December 2021 PubMed Platform: NCBI 1920–December 2021
Search strategy:	CAB Direct: (intensive* OR indoor) AND (farm* OR rear*) pig* OR porcine* OR swine* OR weaner* OR grow* enrich* OR ball OR hanging OR novel OR straw tail biting OR tail-biting 1 AND 2 AND 3 AND 4 PubMed: (intensive OR indoor) AND (farm OR rear) pig OR porcine OR swine OR weaner OR grow enrich OR ball OR hanging OR novel OR straw tail biting OR tail-biting #1 AND #2 AND #3 AND #4
Dates searches performed:	28 Dec 2021

**Exclusion / Inclusion Criteria:** 

Exclusion:	Duplicates. Did not address the PICO question. Enrichment used was not straw.
Inclusion:	Control group. Straw enrichment used. Full text available and in English. Addressed the PICO question.

**Search Outcome:** 

Database	Number of results	Excluded – Did not address PICO question	Excluded – Duplicates	Excluded – Enrichment used was not straw	Excluded – No control group to contrast between with and without enrichment	Total relevant papers
CAB Abstracts	21	14	0	3	1	3
PubMed	5	0	5	0	0	0
Total relevant papers when duplicates removed						3

## Conflict of interest

The authors declare no conflicts of interest.
